# A decomposition of Fisher’s information to inform sample size for developing or updating fair and precise clinical prediction models — part 2: time-to-event outcomes

**DOI:** 10.1186/s41512-025-00204-9

**Published:** 2025-12-16

**Authors:** Richard D. Riley, Gary S. Collins, Lucinda Archer, Rebecca Whittle, Amardeep Legha, Laura Kirton, Paula Dhiman, Mohsen Sadatsafavi, Nicola J. Adderley, Joseph Alderman, Glen P. Martin, Joie Ensor

**Affiliations:** 1 Department of Applied Health Sciences, School of Health Sciences, College of Medicine and Health, University of Birmingham, Birmingham, UK; 2 National Institute for Health and Care Research (NIHR) Birmingham Biomedical Research Centre, Birmingham, UK; 3 Nuffield Department of Orthopaedics, Rheumatology and Musculoskeletal Sciences, Centre for Statistics in Medicine, University of Oxford, Oxford, OX3 7LD UK; 4 Cancer Research UK Clinical Trials Unit, Institute of Cancer and Genomic Sciences, College of Medical and Dental Sciences,, University of Birmingham, Birmingham, UK; 5 Respiratory Evaluation Sciences Program, Faculty of Pharmaceutical Sciences, The University of British Columbia, Vancouver, Canada; 6 Institute of Inflammation and Ageing, College of Medicine and Health, University of Birmingham, Birmingham, UK; 7 Queen Elizabeth Hospital, University Hospitals Birmingham NHS Foundation Trust, Birmingham, UK; 8 Division of Informatics, Imaging and Data Science, Faculty of Biology, Medicine and Health, Manchester Academic Health Science Centre, University of Manchester, Manchester, UK

## Abstract

**Background:**

When developing a clinical prediction model using time-to-event data (i.e. with censoring and different lengths of follow-up), previous research focuses on the sample size needed to minimise overfitting and precisely estimating the overall risk. However, instability of individual-level risk estimates may still be large.

**Methods:**

We propose using a decomposition of Fisher’s information matrix to help examine and calculate the sample size required for developing a model that aims for precise and fair risk estimates. We propose a six-step process which can be used either before data collection or when an existing dataset is available. Steps 1 to 5 require researchers to specify the overall risk in the target population at a key time-point of interest: an assumed pragmatic ‘core model’ in the form of an exponential regression model, the (anticipated) joint distribution of core predictors included in that model and the distribution of censoring times. The ‘core model’ can be specified directly or based on a specified C-index and relative effects of (standardised) predictors. The joint distribution of predictors may be available directly in an existing dataset, in a pilot study or in a synthetic dataset provided by other researchers.

**Results:**

We derive closed-form solutions that decompose the variance of an individual’s estimated event rate into Fisher’s *unit* information matrix, predictor values and total sample size; this allows researchers to calculate and examine uncertainty distributions around individual risk estimates and misclassification probabilities for specified sample sizes. We provide an illustrative example in breast cancer and emphasise the importance of clinical context, including any risk thresholds for decision-making, and examine fairness concerns for pre- and postmenopausal women. Lastly, in two empirical evaluations, we provide reassurance that uncertainty interval widths based on our exponential approach are close to using more flexible parametric models.

**Conclusions:**

Our approach allows users to identify the (target) sample size required to develop a prediction model for time-to-event outcomes, via the pmstabilityss module. It aims to facilitate models with improved trust, reliability and fairness in individual-level predictions.

**Supplementary Information:**

The online version contains supplementary material available at 10.1186/s41512-025-00204-9.

## Background

Studies developing a clinical prediction model use a sample of data, ideally representative of a target population (e.g. women diagnosed with breast cancer), to produce a model (e.g. regression, random survival forest or deep learning) for estimating an individual’s risk of a particular outcome (e.g. 5-year risk of breast cancer recurrence) conditional on their values of multiple predictors (covariates, features). An example is the ‘post D-dimer’ model of Ensor et al. to estimate an individual’s risk (by 1, 2 or 3 years) of a recurrent venous thromboembolism event following cessation of therapy for a first event. [1]

When developing a prediction model, there is a responsibility to implement rigorous standards in study design and analysis [2–4]. An important design aspect, which is often neglected [5–12], is the sample size requirements to develop and evaluate these models. In previous work, we outlined how to calculate the minimum sample size needed for model development for time-to-event outcomes [13], based on (i) estimating the overall event risk by a particular time-point precisely and (ii) minimising model overfitting for a regression-based prediction model in terms of overall fit and population-level calibration slope. These criteria are implemented in the Stata and R module *pmsampsize *[14, 15], and they aim to target a reliable prediction model at least at the population level, corresponding to the first two model stability levels defined by Riley and Collins [16].

As clinical prediction models are used to guide *individual *decision-making, targeting reliable predictions at the subgroup and individual level is important, corresponding to the third and fourth stability levels defined by Riley and Collins [16]. This may require considerably larger sample sizes than the minimum recommended by previous approaches targeting the population level, for example to ensure precise risk estimates at the individual level that correspond to sufficiently narrow uncertainty distributions and intervals for an individual’s ‘true’ risk. In this article, we derive sample size calculations for developing prediction models using time-to-event outcome data (i.e. when participants contribute different lengths of follow-up due to censoring), where the aim is precise individual-level estimates of event risk by one or more time-points of interest. This extends our work for binary outcomes [16, 17]. We derive closed-form solutions based on decomposing prediction variances into Fisher’s unit information matrix and total sample size, which can be used (either before data collection or when an existing dataset is available) to examine how sample size impacts uncertainty distributions and intervals around individual-level risk estimates from a model with a pre-specified core set of predictors. The approach can be used either before data collection or when an existing dataset is available, to help researchers identify a suitable sample size (before any model building) that targets precise individual-level risk estimates for informing patient counselling, enhancing clinical decision-making and ensuring fairness [18].

The article outline is as follows. We begin by summarising our existing sample size approach targeting precise population-level predictions. Then, we outline our new proposal to examine sample size requirements to target precise and fair individual-level predictions, based on analytic solutions assuming an exponential survival model. We also show how this allows the probability of misclassification to be examined in situations where risk thresholds are used for clinical decision-making. In our Results sections, we apply our proposal to two clinical examples, which includes an illustration of fairness investigations, and also empirically compares the exponential modelling assumption to Weibull and flexible parametric models. We then conclude with discussion.

## Existing sample size approach to precisely estimate overall risk and minimise overfitting for a time-to-event outcome

Our previously proposed approach calculates the minimum required sample size for prediction model development [13, 19], to meet the following criteria:

- Criterion (i): A precise estimate of the overall outcome risk by a particular time-point (e.g. absolute margin of error ≤ 0.05)
- Criterion (ii): Small overfitting of predictor effects defined by an overall shrinkage factor ≥ 0.9
- Criterion (iii): Small optimism in apparent model fit defined by Nagelkerke R-squared $\left(R_{Nagelkerke}^2\right)$, such as ≤ 0.05

Details of the calculations are provided in our previous papers [13, 19]. The approach is implemented in the Stata and R modules *pmsampsize *[14, 15], with the user needing to specify the overall outcome risk (prevalence) at the time-point of interest, the anticipated model performance in the target population (quantified by ${R}_{Nagelkerke}^{2}$, Cox-Snell R-squared (${R}_{CS}^{2}$), or the C-index) and the number of candidate predictor parameters for the model. For example, consider developing a model to estimate the 5-year recurrence risk of breast cancer, based on a core set of six predictor parameters and assuming a ${R}_{CS}^{2}$ of 0.14 (corresponding to a C-index of 0.70), with a mean follow-up of 3.57 years and event rate of 0.099 recurrences per person year. Applying our previous approach via *pmsampsize* suggests a minimum of 355 participants are required for model development, with about 126 recurrences during 1268 person-years of follow-up.

## Methods: a new sample size approach to target precise estimates of individual risk and improve model fairness

We now introduce a six-step approach to examine the sample size required for model development to target sufficiently precise individual risk estimates at a particular time-point of interest. The process requires specifying the joint distribution of a core set of predictors and an assumed exponential survival model. A closed-form decomposition of Fisher’s unit information matrix is then used to derive anticipated uncertainty intervals for a particular sample size. This is implemented using our accompanying *pmstabilityss* module in Stata (see code at https://github.com/JoieEnsor/pmstabilityss-TTE), with R and Python versions forthcoming.

### Step-by-step guide to the proposal sample size calculation

#### Step (1) — Identify a core set of predictors, variables linked to fairness and a key time-point of interest

The precision of risk estimates at the individual level depends on the (joint) distribution of predictors (features) that will be included in the developed model. Therefore, to inform the sample size calculation, the first step is to identify a set of *core predictors*; that is, clinical variables well-known to contribute important key predictive information. Core predictors (e.g. age and stage of disease for cancer outcome prediction) can be identified from previously published models, systematic reviews of prognostic factor studies [20] and conversations with clinical experts. Though additional potential predictors might be considered during the actual model development, the premise is that these additional variables typically add little or no predictive information beyond the core predictors and may even increase instability and imprecision. In other words, the set of core predictors represent a fundamental basis for targeting precise individual-level risks and define the bare minimum for which we want to control (in)stability.

Further to the core predictors, it may be important to also identify variables linked to fairness checks. For example, regardless of whether they are included in the developed model, variables such as age, sex, ethnicity and others (e.g. those representing protected characteristics and subgroups) might be identified so that ultimately (see Step (6)) the researcher can examine the sample size required to target sufficiently precise predictions for each subgroup.

Step (1) should also identify a key time-point of interest for prediction, that is, the time-point for which the model will estimate the risk of having the outcome event by. If multiple time-points are relevant, then the sample size calculation should be repeated for each time-point, as the precision of a risk estimate depends on the time-point chosen. We focus on one key time-point here onwards.

#### Step (2) — Obtain a dataset that reflects the joint distribution of the core predictors

The joint distribution of predictors that are included in the final prediction model impacts the standard errors of the model parameter estimates (e.g. intercept or predictor effects in a time-to-event regression model) and influences the width of uncertainty intervals around individual-level risk estimates. Hence, step (2) requires the user to specify the joint distribution of core predictors selected in step (1) and obtain or create a (synthetic) sample of data that reflects this. How to achieve this will depend on the availability of the model development dataset, as follows:

- *Dataset is already available for model developers*: This is the most common and simple scenario, as the joint distributions of predictors (and event/censoring times — see step (4)) are already observed. Thus, the user does not need to do anything in this step, as this existing dataset can be used directly in subsequent steps where needed (e.g. in step (5) to derive the unit information matrix).
- *Dataset exists but not yet available for model developers (e.g. access to a previously collected dataset is conditional on funding success)*: The data holders could be contacted and asked to provide a synthetic dataset that mimics the joint predictor and event (censoring) time distributions, for instance as obtained by a simulation-based approach that models conditional relationships [21], using packages such as *synthpop* in R [22]. Later, we showcase this approach, but another notable example is the Clinical Practice Research Datalink (CPRD), which has generated synthetic datasets to aid researchers improve workflows (https://www.cprd.com/synthetic-data). Alternatively, data holders could provide summary details of the joint distributions (e.g. cross-tabulations of categorical variables; variance-covariance matrix of continuous variables), to allow the user themselves to simulate a large synthetic dataset containing predictor values for, say, 10,000 individuals. Previous studies that already use the dataset of interest may have summarised baseline variables (e.g. means, standard deviations (SDs), proportions) in their articles, for example within baseline characteristics table, though without covariances.
- *New data collection required (e.g. a planned prospective cohort study)*: When new data are required, the joint predictor distribution could be based on summary information (e.g. means and SDs for continuous predictors, proportions in each group for categorical predictors) from previous studies in the same target population, for example using published tables of baseline characteristics. These can then be used to simulate a large synthetic dataset of predictor values, as detailed above. A challenge is that often only the marginal distribution for each predictor will be summarised (e.g. from published tables of baseline characteristics), and the correlation or conditional relationship amongst predictors will be unknown. In this situation, a pragmatic starting point is to assume the predictors are conditionally independent, but the impact of this should be examined through sensitivity analyses.

#### Step (3) — Specify a ‘core model’ for how individual risks depend on core predictor values

Alongside an individual’s predictor values, the uncertainty around an individual’s risk estimate at a particular time-point depends on their underlying event rate as a function of time. Therefore, the user should specify a model that expresses how an individual’s event rate over time depends on the values of core predictors from step (2) (referred to as the ‘core model’). For example, a parametric time-to-event regression model could be specified, such that the log event rate is a function defined by a baseline log hazard function and beta (log-hazard ratio) terms, which are chosen to match the overall risk at the time-point of interest (e.g. from a Kaplan–Meier curve) and core predictor effects from previous studies in the same population. Often, the ‘core model’ will be based on an existing model where the aim is to extend a previously published model (e.g. by adding more predictors) or update all parameters of an existing model (e.g. due to concerns of calibration drifts affecting intercept and predictor effect estimates).

The simplest, and thus most practical approach, is to assume the ‘core model’ is an exponential proportional hazards regression model, such that each participant $i$ has a constant hazard rate of ${\eta }_{i}$ and event times (${t}_{i}$) are exponentially distributed conditional on values of $\text{P}$ core predictors. This model can be written as follows:

1 $$\begin{aligned} &t_i \sim\text{exponential}(\eta_i)\\ &\text{ln}\left(\eta_i\right) = \mu_i=\alpha+\beta_1x_{1i}+\beta_2x_{2i}+\dots+\beta_\text{P}x_{\text{P}i}\end{aligned}$$

Apart from the intercept $\alpha$ (which defines the constant baseline hazard), all parameters correspond to log rate (hazard) ratios, quantifying the change in log rate for a one-unit increase in the corresponding $x$ value. Equation (1) can equivalently be written as an accelerated failure time (AFT) model, as follows: [23]

2 $${\text{ln}\;(t}_i)=-\mu_i+\varepsilon_i=-\alpha-\beta_1x_{1i}-\beta_2x_{2i}-\dots-\beta_\text{P}x_{\text{P}i}+\varepsilon_i$$

where ${\varepsilon }_{i}$ follows an extreme value distribution with probability density function (pdf) ${f(\varepsilon }_{i})=\text{exp}({\varepsilon }_{i})\text{exp}(-\text{exp}{(\varepsilon }_{i}))$. We will use this AFT specification when deriving the variance matrix of parameter estimates in step (5), but the interpretation of parameters is identical to before, and each patient contributes data ${{\varvec{x}}}_{{\varvec{i}}}=(1,{x}_{1\text{i}},{x}_{2\text{i}},\dots ,{x}_{\text{Pi}})$. Censoring is considered in steps (4) and (5).

#### Rationale for choosing an exponential distribution

The exponential model is a pragmatic choice: assuming a constant baseline hazard rate ($\alpha )$ makes the ‘core model’ specification (and thus the whole sample size calculation) easiest for researchers. The approach also results in equivalent specification of the model in terms of both proportional hazards and AFT formats, thus providing a common basis for both types of models. In many situations, a nonconstant (potentially even non-linear) baseline hazard rate might be preferable, for example as defined by a parametric distribution such as a Weibull, log-normal or gamma distribution. However, this ultimately creates complexity for providing analytic sample size solutions for the uncertainty of risk estimates, because the more complex parametric distributions require extra (ancillary) parameters than those for the exponential model (e.g. shape and scale parameters for a Weibull model). The uncertainty of these ancillary parameters needs to be accounted for when deriving uncertainty intervals for prediction, which is challenging as predictions depend on a complex (non-linear) function of ${\mu }_{i}$ and the ancillary parameters. Although this is achievable post model fitting, here we are focused on sample size calculations before any data analysis and so use the exponential model, as it provides analytic solutions that are closed form and disentangle sample size from other aspects (see step (6)). We note that, for the same reason, analytic (i.e. not simulation-based) sample size calculations for survival data in other settings (e.g. randomised trials evaluating treatment effect) often assume exponential and proportional hazards for simplicity to produce closed-form solutions. Later, we empirically evaluate the impact of assuming the exponential distribution compared to using Weibull or flexible parametric models.

#### Approaches for specifying the ‘core model’

A ‘core model’ may be difficult to specify; for example, predictor effects may not be readily available if previous models were incompletely reported. We address this by outlining two approaches to simplify the process, utilising the anticipated C-index of the model and overall risk (for the time-point of interest, defined in step (1)) in the target population, alongside assumptions of relative predictor effects (weights).

- Approach (a): Specify the overall risk at a particular time-point, C-index and relative weights of core predictors. With this information, we can use an iterative approach to identify an exponential regression equation forming a ‘core model’ consistent with the specified overall risk and C-index whilst retaining the relative weight of core predictors. This approach simulates predictor values for a large number of participants (based on the distributions specified in step (2)) and then uses an iterative process to identify values of the intercept $(\alpha)$ and a multiplicative factor $\left(\delta\right)$ of the following model:

3 $$\begin{aligned} &t_i\sim\text{ exponential}(\eta_i)\\ &{\text{ln}\left(\eta_i\right)=\mu}_i=\alpha+\delta\left(\beta_1x_{1i}+\beta_2x_{2i}+\dots+\beta_\text{P}x_{\text{P}i}\right) \end{aligned}$$

where beta coefficients are the user-specified relative weights. Convergence is achieved when the model reaches the specified C-index and overall risk within a small margin of error. Often, Harrell’s C-index is provided, but other time-dependent C statistics could also be used as needed.

To simplify the specification of relative weights, we can rescale variables so that relative effects are the same. Specifically, continuous variables could be rescaled (e.g. age specified in 10 years) so the effect (log-hazard ratio) of a one-unit increase (e.g. 10-year increase) is considered to have the same predictive effect as a one-unit increase in other variables (e.g., smoker versus non-smoker). More broadly, standardisation could be used as follows in approach (b).

Recall that core predictors in step (2) may include protected characteristics, even if they do not have any predictive effect. If the latter, their relative weight can be set to zero in the ‘core model’. Nevertheless, having them in the synthetic or existing dataset ultimately allows prediction and classification uncertainty to be assessed for them.

- Approach (b): Specify the overall risk and C-index whilst assuming same weight of predictors on their standardised scale. This approach standardises each core predictor (i.e. uses $((x_{1i} - \bar{x}_{1i})/SD_{x_{1i}},(x_{2i} - \bar{x}_{2i})/SD_{x_{2i}})$ etc) from step (2), and then the ‘core model’ of Eq.(3) is specified in terms of these standardised predictors and sets all beta weights to be equal (e.g. = 1). Essentially, this approach assumes the predictive effect of a 1-SD increase to be the same for each standardised predictor. As in approach (a), the iterative approach is then used to identify $\alpha$ and $\delta$ that ensure the ‘core model’ has the specified C-index and overall risk.

#### Step (4) — Derive outcome event times and censoring times

As the variance of predictions depends on observed event and censoring times (see step (5)), they need to be available alongside the predictor values. If an existing (full or pilot) or synthetic dataset is available (see step (2)), the outcome event or censoring time can be observed directly for each individual. Otherwise, step (4) requires outcome event times to be generated in the large dataset conditional on the assumed ‘core model’, for example using the *simsurv* package in R or Stata [24, 25], which allows the user to specify the survival distribution (e.g. exponential) and predictor effects. Similarly, unless existing or synthetic data are available, the censoring times need to be generated from an assumed survival model of the censoring rate, which usually will be assumed independent of predictors and outcome risk for simplicity. Without other knowledge, it is pragmatic to assume an exponential distribution with a constant censoring rate. Lastly, for each individual, define ${y}_{i}$ as the minimum of their observed log event time and log censoring time (of course, when using a real dataset, only one of these is observed anyway).

#### Step (5) — Derive Fisher’s unit information after decomposing Fisher’s information matrix

Steps (5) and (6) involve approximating (based on the information from steps (1) to (4)) the anticipated variance–covariance matrix ($\text{var}(\widehat{\boldsymbol\beta})$) of model parameter estimates ($\widehat{\boldsymbol\beta}={(\widehat\alpha,\widehat\beta}_1,{\widehat\beta}_2,\dots,{\widehat\beta}_\text{P})')$ if we were to fit the assumed ‘core model’ for a particular sample size. This is needed, as the variance–covariance matrix of the parameters in the ‘core model’ is subsequently used to calculate the variance of individual-level risk estimates at the time-point of interest.

The key mathematical foundation for step (5) is to recognise that $$\text{var(}\widehat{\boldsymbol\beta})$$ (the inverse of Fisher’s information matrix) can be decomposed into the total sample size ($$n)$$ and Fisher’s *unit* information matrix ($$\mathbf{I}$$):

4 $$\text{var(}\widehat{\boldsymbol\beta})=n^{-1}\mathbf I^{-1}$$

The unit information matrix is defined by the following:

5 $$\begin{aligned} \mathbf {I} &=E\left(\mathbf{X}^{\boldsymbol{\prime}}\mathbf{X}\exp\left(y_i+{\widehat\mu}_i\right)\right)\\ &= E(\mathbf{X}^{\boldsymbol{\prime}}\mathbf{X}\exp(w_i))\\ & = E(\mathbf A) \end{aligned}$$

where $\mathbf{X}$ is the design matrix for the assumed ‘core model’, with each individual’s data corresponding to ${{\varvec{x}}}_{{\varvec{i}}}=(1,{x}_{1\text{i}},{x}_{2\text{i}},\dots ,{x}_{\text{Pi}})$. The $E\left(\mathbf{A}\right)$ is the expected value of matrix $\mathbf{A}$ and depends on the joint distribution of the predictors (as this defines $\mathbf{X}$), the parameter values of the ‘core model’ (as this defines ${\widehat{\mu }}_{i}$) and the observed log follow-up time $({y}_{i})$ for each participant (i.e. the minimum of their log event time and log censoring times)[26]. The latter adds complication due to the censoring inherent within time-to-event data (hence step (4)).

A simple way to derive $E\left(\mathbf{A}\right)$ is to calculate each of the components of $\mathbf{A}$ for each participant in the (existing or simulated) dataset from step (2), using each participant’s observed predictor values and observed event or censoring time (${y}_{i}$), combined with the (exponential) regression parameters of the ‘core model’ from step (3); the means (across all participants) of each component then provide their expected values and form $\mathbf{I}$. Our module *pmstabilityss* module implements this.

For example, assuming three core predictors and specifying our ‘core model’ as the following exponential regression:

$$\begin{aligned} &{t}_{i} \sim \text{ exponential}\left({\eta }_{i}\right)\\ &{\text{ln}\left({\eta }_{i}\right)=\mu }_{i}=\alpha +{\beta }_{1}{x}_{1i}+{\beta }_{2}{x}_{2i}+{\beta }_{3}{x}_{3i}\end{aligned}$$

Then $\mathbf{X}^{\boldsymbol{\prime}}\mathbf{X}$ is a 4 × 4 matrix due to the four parameters in the regression equation ($\alpha$, ${\beta }_{1}$, ${\beta }_{2}$, ${\beta }_{3}$), where $\mathbf{X}$ is the design matrix for the assumed ‘core model’, with each individual’s data corresponding to ${{\varvec{x}}}_{{\varvec{i}}}=(1,{x}_{1\text{i}},{x}_{2\text{i}},{x}_{3\text{i}})$. This implies that as follows:

$$\mathbf I=E\left(\mathbf X'\mathbf X\exp\left(y_i+{\widehat\mu}_i\right)\right)=E\left(\mathbf X'\mathbf Xw_i\right)$$

$$={ E}_{\left(x,w\right)}\left[\begin{array}{cccc}{w}_{i}& {x}_{1i}{w}_{i}& {x}_{2i}{w}_{i}& {x}_{3i}{w}_{i}\\ {x}_{1i}{w}_{i}& {x}_{1i}^{2}{w}_{i}& {x}_{1i}{x}_{2i}{w}_{i}& {x}_{1i}{x}_{3i}{w}_{i}\\ {x}_{2i}{w}_{i}& {x}_{1i}{x}_{2i}{w}_{i}& {x}_{2i}^{2}{w}_{i}& {x}_{2i}{x}_{3i}{w}_{i}\\ {x}_{3i}{w}_{i}& {x}_{1i}{x}_{3i}{w}_{i}& {x}_{2i}{x}_{3i}{w}_{i}& {x}_{3i}^{2}{w}_{i}\end{array}\right]$$

where $${w}_{i}=\text{exp}\left({y}_{i}+{\widehat{\mu }}_{i}\right)$$, $${\widehat{\mu }}_{i}$$ is an individual’s estimated linear predictor value from the fitted exponential regression (e.g., $${\widehat{\mu }}_{i}={\widehat{\alpha }}_{i}+{\widehat{\beta }}_{1}{x}_{1i}+{\widehat{\beta }}_{2}{x}_{2i}+{\widehat{\beta }}_{3}{x}_{3i}$$), and $${y}_{i}$$ is an individual’s log follow-up time (i.e. minimum of the log event time and log censoring time for each patient$$)$$. We derive the anticipated $$\mathbf{I}$$ (automated in *pmstabilityss*) as follows:

- Set the parameter values in the ‘core model’ (i.e. $\alpha$, $\delta$, and all $\beta$) at their anticipated true values, defined in step (3).
- Set each individual’s $x_{1i}$, $x_{2i}$, and $x_{3i}$ values as the (standardised or unstandardised) observed values of core predictors in the (existing or synthetic) dataset from step (2).
- Use this information to derive $\mathbf{X}$ and $\exp\left(y_i+\widehat{\mu_i}\right)$
- Derive $E\left(\mathbf A\right)$ by calculating the 16 components of $\boldsymbol A$ for each participant in the (existing or synthetic) dataset, and then the mean of each component provides their expected values and thus forms $\mathbf{I}$.

#### Step (6) — Examine the impact of sample size on precision of individual risk estimates

The final step is to examine how sample size impacts the level of precision (uncertainty distribution and interval widths) around individual risk estimates at the key time-point of interest (as chosen in step (1)). This is relevant when the user has an existing dataset (to check if this sample size is large enough) or when designing a new study with prospective data collection (to a priori identify the required sample size). These situations are now outlined as Options A and B below.

- Option A: Calculate expected uncertainty of predictions for a given sample size (existing dataset)

Following maximum likelihood estimation of an exponential regression model, the variance of a new individual’s log (hazard) rate (${\widehat{\mu }}_{new}$) is as follows:

6 $$\text{var}\left(\widehat{\upmu}_{{new}}\right) = \text{var}\left(\mathbf{x}_{{new}}\left(\widehat{\boldsymbol{\upbeta}}\right)\right) = \mathbf{x}_{{new}}\text{var}\left(\widehat{\boldsymbol{\upbeta}}\right)\mathbf{x}^{\boldsymbol{\prime}}_{{new}}$$

where ${{\mathrm{x}}}_{{{n}}{{e}}{{w}}}=(1,{x}_{1new},{x}_{2new},\dots ,{x}_{\text{P}new})$ are the predictor values for the new individual. Substituting in Eq. (4), this can be rewritten as follows:

7 $$\text{var}\left(\widehat{\upmu}_{{new}}\right) = \text{{n}}^{-1}\ \mathbf{x}_{{new}}\ \mathbf{I}^{\boldsymbol{-1}}\ \mathbf{x}^{\boldsymbol{\prime}}_{{new}}$$

Hence, for a specified sample size $(n)$, we can derive an anticipated 95% uncertainty interval around an individual’s log rate using the following:

8 $$\left[{\widehat{\mu }}_{new}\pm \left(1.96\times \sqrt{\text{var(}{\widehat{\mu }}_{new})}\right)\right]= \left[\widehat{\mu}_{new\_L},\widehat{\mu }_{new\_U}\right]$$

where $$L$$ and $$U$$ denote lower and upper, respectively. However, the actual scale of interest is the survival probability scale ($$S(t)$$) or the event risk scale ($$F\left(t\right)=1-S\left(t\right)$$). Focusing on the latter, we can derive an individual’s true event risk (based on the ‘core model’) at a particular time-point ($$t)$$ using the following:

9 $$F_{new}\left(t\right)=1-\left(\text{exp}\left(-\text{exp}\left(\mathbf{x}_{new}\widehat{\boldsymbol{\upbeta}}\right)\right)t\right)$$

with $\widehat{\boldsymbol{\upbeta}}$ replaced by the true parameters of the core model. Then, the anticipated 95% uncertainty interval for an individual’s event risk at $t$ is given by the following:

10 $$\left[1-\text{exp}\left(-\text{exp}\left(\widehat{\mu}_{new\_L}\right)\right)t), 1-\text{exp}\left(-\text{exp}\left(\widehat{\mu}_{new\_U}\right)\right)t)\right]$$

As this is a frequentist framework, this interval is akin to a 95% confidence interval for the individual’s ‘true’ risk. A more formal interpretation of the confidence interval is as follows: if we repeated the model development study many times (with the same sample size, set of predictors and regression framework) and use Eq. (10) to derive a 95% interval each time, then 95% of the intervals we create are expected to contain the individual’s ‘true’ risk.

Hence, option A requires the user to calculate Eq. (7) to Eq. (10) and derive uncertainty intervals for individual risk for each participant (or at least those within key subgroups) in the target population, conditional on a specified model development sample size ($n$) and a time-point ($t$) of interest. The time-point was already chosen in step (1); the unit information matrix ($\mathbf{I}$) from step (5) and the participants of interest in the target population can just be those from the existing or simulated dataset from step (2) which already contain predictor values ($\mathbf{x}_{new}$). Thus, to apply Eq. (7) to Eq. (10), all that remains is to specify the sample size of interest; this could be the available number of participants in the existing dataset being considered for model development, or it might be a specified sample size being considered for new data collection (e.g. that determined by *pmsampsize*). Sometimes, a range of different sample sizes might be considered to examine the value of information (e.g. in terms of reduced width of uncertainty intervals, reduced classification instability) arising from including additional participants over and above that recommended by *pmsampsize*.

More generally, based on maximum likelihood theory, we could assume that approximately $\widehat{\boldsymbol\beta}\sim N(\boldsymbol\beta,\text{var}\left(\widehat{\boldsymbol\beta}\right))$ and use this to sample (e.g. 1000) potential parameter estimates for a chosen sample size; these can then be used to derive a sample of potential risk estimates for each new individual using Eq. (9), to reflect their uncertainty distribution of risk.

- Option B: Calculate a target sample size for new data collection to ensure precise individual-level predictions

When designing a new study to recruit participants for model development, researchers will need to calculate the sample size required to target particular precision of risk estimates. By rearranging Eq. (7), the sample size needed to target a chosen variance of the log-rate estimate for an individual is as follows:

11 $${n}= \mathrm{var}\left(\widehat{\mu}_{{new}}\right)^{-1} \mathbf{x}_{{new}}\; \mathbf{I}^{\boldsymbol{-1}}\;\mathbf{x}^{\boldsymbol{\prime}}_{{new}}$$

Equation (11) can then be applied to each individual in the (real or simulated) dataset from step (2) to obtain the required $n$ for their particular combination of predictor values (${{\mathrm{x}}}_{new})$. A practical issue is how to select the target value of ${\text{var}}\left({\widehat{\mu }}_{new}\right)$ for each individual, as this is on a difficult scale to interpret. Further, the required value of ${\text{var}}\left({\widehat{\mu }}_{new}\right)$ will not be consistent across individuals, due to the multiplication with $\mathbf{x}_{{new}}\; \mathbf{I}^{\boldsymbol{-1}}\;\mathbf{x}^{\boldsymbol{\prime}}_{{new}}$, which is individual specific. A pragmatic approach is to specify the maximum ${\text{var}}\left({\widehat{\mu }}_{new}\right)$ allowed for a range of ${F}_{new}\left(t\right)$ values (e.g. 0.01, 0.025, 0.05, 0.10, 0.15, 0.20), corresponding to a target maximum uncertainty interval width on the event risk (i.e. ${F}_{new}\left(t\right))$ scale via Eq. (9) and Eq. (10)). These can then be applied to each individual by using the ${\text{var}}\left({\widehat{\mu }}_{new}\right)$ value that corresponds to the categorised ${F}_{new}\left(t\right)$ value closest to their true ${F}_{new}\left(t\right)$. Special attention may also be given to selecting appropriate ${\text{var}}\left({\widehat{\mu }}_{new}\right)$ values in particular subgroups defined by combinations of predictor values (e.g. sex, ethnicity), where algorithmic fairness checks will be important.

#### Deciding and presenting target uncertainty intervals with patients and clinical stakeholders: perspective based on risk thresholds and decision theory

Regardless of whether option A or B is chosen, model developers will need to decide what width of uncertainty intervals they deem appropriate. Ideally, a suitably narrow interval is desired for *every* individual (for all combinations of values of predictors in the ‘core model’). However, depending on the clinical context and role of the model for clinical practice, some regions of estimated risk may not require intervals to be as narrow as in other regions. For instance, having wide uncertainty intervals for individuals with high risk (e.g. reflected by uncertainty intervals from 0.3 to 0.95) may not matter (i.e. medical decisions would be the same) if the entire interval range is compatible with a perceived high risk. This concept aligns with preferences for risk thresholds for clinical decisions. For example, a 10-year CVD risk threshold of 0.1 (i.e. 10%) is typically used to guide decisions to prescribe statins, and so wide uncertainty intervals that span 0.3 to 0.95 might be deemed acceptable, but narrower intervals that span 0.05 to 0.3 (including the threshold of 0.1) may not. When deciding on appropriate uncertainty interval widths, it is important to understand the clinical context of how the model will be used to guide decision-making and any corresponding risk threshold(s) involved.

With this in mind, it will be helpful to identify risk thresholds from a decision-theory perspective [27–29], based on preferences (utilities) elicited from patients, clinicians and other relevant stakeholders about particular outcomes and consequences that may follow from possible decisions. For example, a VTE 3-year recurrence risk threshold of about 5% is suggested for when an individual should be recommended to continue anti-coagulation treatment [1]. If a well-calibrated prediction model estimates the individual’s risk to be $\ge$5%, then this suggests the correct decision is to continue anti-coagulation treatment. However, there may still be uncertainty about the suggested decision due to uncertainty of the model’s risk estimate; if the uncertainty is too wide, then ideally additional information is needed before making a decision [30].

In this context, the aim of our sample size approach is to help understand and examine which sample sizes are likely to give sufficient information to guide decisions at the individual level. To help examine this, we recommend calculating and presenting to stakeholders as follows:

- Prediction instability plots, where each individual’s ‘true’ risk from step 3 (x-axis) is plotted against their corresponding uncertainty interval (y-axis) from step 5. The question to ask stakeholders is whether the individual uncertainty intervals are too wide for using or endorsing the model in practice. To facilitate this discussion, we recommend prediction instability plots are presented with two curves (e.g. using a LOWESS smoother or spline function) fitted separately through individuals’ upper and lower uncertainty interval values. These curves define a ‘typical’ 95% uncertainty interval at each risk, across the entire spectrum of estimated risks from 0 to 1, which should aid visual interpretation for stakeholders (as individual uncertainty intervals can vary considerably, even for those with the same estimated risk). This is demonstrated later in the article.
- Classification instability plots (if risk thresholds are relevant), plotting each individual’s ‘true’ risk (x-axis) (i.e. the risk defined by the ‘core model’) against the proportion (y-axis) of their uncertainty distribution that falls on the opposite side of their chosen clinical risk threshold compared to their ‘true’ risk. The question to ask stakeholders is whether, in general, the proportion of the uncertainty distribution on the opposite side of the threshold is generally too large for them to use or endorse the model for individuals in practice. In our examples later, we assume the same risk threshold is relevant for all individuals, but this can be relaxed if stakeholders recommend different thresholds across particular subgroups.
- Summary statistics, which quantify the magnitude of uncertainty and classification instability across individuals. In particular, report the mean (min, max) width of 95% confidence intervals, and the mean (min, max) probability of misclassification. Also, report the mean (min, max) across individuals of their mean absolute prediction error (MAPE), or root-mean-squared prediction error (RMSPE), which can be derived by many (e.g., 1000) sampling values from each individual’s uncertainty distribution and calculating mean absolute (or root mean-squared) differences to their ‘true’ risk.
- Subgroup plots and results, which summarise the anticipated uncertainty and classification instability in relevant subgroups of people (e.g. defined by sex, ethnicity — see example later.

We now illustrate all these ideas with two examples applying our new approach. In the supplementary material S1, we also discuss how to measure the impact of uncertainty on clinical utility, using the net benefit function [31].

## Results I: Application to a model for breast cancer recurrence by 5 years

Consider the scenario where researchers want to develop a prediction model for estimating the risk of breast cancer recurrence within 5 years in those with node-positive and oestrogen-receptor (ER)-positive breast cancer treated with tamoxifen. They plan to recruit participants to a new cohort study and want to know the sample size required to produce a reliable model. Using *pmsampsize* and assuming a mean follow-up of 3.57 years and event rate of 0.099 recurrences per person year, a minimum of 355 participants is recommended to identify the sample size needed to target population-level stability in estimated risks.

As the model is to be used to guide individual-level counselling and decision-making, it is also important to target precise estimation of individual-level risk estimates. This can be done using the six-step process outlined in the previous section, and we now describe this process, under three scenarios. Initially, we assume that an existing dataset is available to inform the calculations. Then we consider that in the absence of an existing dataset, a synthetic dataset has been generated and provided by external researchers, firstly with and then without follow-up information included.

### Sample size calculation based on an existing dataset at hand

A previous dataset is available of 686 patients diagnosed with node-positive breast cancer from 1984 to 1989 and recruited to the German Breast Cancer Study Group (GBSG). The GBSG dataset contains five core predictors considered important to predict 5-year survival (see step 1) alongside information about follow-up and any recurrence times [32, 33]. Here, to inform our sample size calculations, we focus on the subset of 220 participants that were ER positive and received tamoxifen treatment, which represents our target population for the new prediction model. Henceforth, we consider this dataset as akin to a pilot study, and *pmstabilityss* uses it within the six-step process described in the previous section to examine required sample sizes.

### Step (1) — Identify a core set of predictors

Five core predictors were identified: age (years), tumour size (mm), number of positive lymph nodes, menopausal status (pre or post) and tumour grade (1, 2 or 3). This corresponds to six predictor parameters. To help with specification of relative weights (see step (3)), the three continuous predictors were standardised (e.g. age was specified as $\left({\text{age}}_{i}-{\overline{\text{age}} }_{i}\right)/{\text{SD}}_{{\text{age}}_{i}}$).

### Step (2) — Specify the joint distribution of the core predictors

The joint distribution of the 5 core predictors was observed in the 220 participants from the GBSG pilot dataset; thus, no synthetic data was needed. Hence, subsequent prediction and instability plots (see step 6) are based on these 220 participants.

### Step (3) — Specify a ‘core model’ for how individual risks depend on core predictor values

The ‘core model’ was chosen to be an exponential proportional hazards model with relative predictor weights identified from analysis of the GBSG dataset itself and, for simplicity, assuming linear functional form for continuous predictors on their standardised scale:

$$\begin{aligned} {t}_{i}\sim \text{e} & \text{xponential}\left({\eta }_{i}\right)\\ \text{ln}\left({\eta }_{i}\right)&=\mu_{i}\\ &=\alpha +\delta \left((-1 \times \text{ age}) + (0.5 \times \text{ size})\right.\\ &\quad + (2 \times \#\text{nodes}) + (3 \times \text{ post}\_\text{menopause})\\ &\quad + (3 \times \text{ grade}2) + (4 \times \text{grade}3))\end{aligned}$$

The $\alpha$ and $\delta$ values were set to be − 3.429 and 0.208, respectively, which ensure that the ‘core model’ had a C-index of 0.70 and a mean estimated risk of 0.39 in the 220 GBSG patients, in order to match the observed performance and overall 5-year recurrence risk in the dataset. Recent survival statistics for ER-positive patients also reported a similar 5-year recurrence risk [34]. For brevity, the impact of changing these predictor weights is considered in supplementary material S3.

### Step (4) — Derive outcome event times and censoring times

The time of either censoring or recurrence was available for each participant in the GBSG dataset; this allowed us to define their ${y}_{i}$ variable (i.e. log follow-up time, representing the minimum of log time of censoring or recurrence). Using the ${\widehat{\mu }}_{i}$ from Step (3), we calculate ${w}_{i}=\text{exp}\left({y}_{i}+{\widehat{\mu }}_{i}\right)$ values toward Fisher’s unit information matrix. The mean follow-up time was 3.57 years across all participants, 4.11 years in those that were censored before recurrence and 2.59 years in those that had a recurrence. The maximum follow-up length was 7.28 years, the total follow-up was 785.4 person-years and the overall event rate was 0.099 (= 78/785.4) recurrences per person year.

### Step (5) — Derive Fisher’s unit information after decomposing Fisher’s information matrix

Apply Eq. (5) to derive the unit information matrix ($\mathbf{I}$).

### Step (6) — Examine the impact of sample size on precision of individual risk estimates

**Fig. 1:**
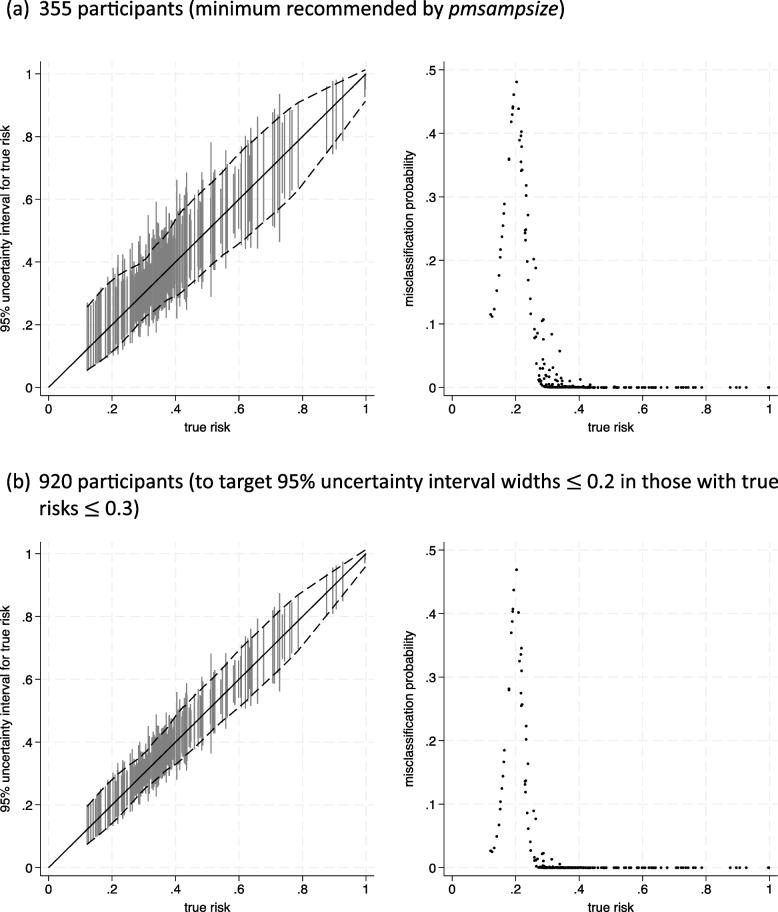
Prediction and classification instability plots for the breast cancer recurrence model assuming a particular sample size, using the GBSG dataset of 220 patients for informing the joint distribution of predictors and censoring and recurrence times (dash lines correspond to 95% uncertainty intervals). Classification is based on a risk threshold of 20% **a** Three-hundred fifty-five participants (minimum recommended by pmsampsize).** b** Nine-hundred twenty participants (to target 95% uncertainty interval widths ≤ 0.2 in those with true risks

We focus on estimates of risk at 5 years and examine how sample size impacts the width of 95% uncertainty intervals around risk estimates. We summarise the probability of misclassification based on an illustrative risk threshold of 20%, for example to identify high-risk individuals for whom chemotherapy might be considered (the impact of using a different threshold is considered in supplementary material S3). We begin by considering the sample size recommended by *pmsampsize*, which was 355 participants (see earlier in the article). Prediction and classification instability plots are shown in Fig. 1a and summary statistics in Table 1.

**Table 1:** Summary statistics for the expected precision and probability of misclassification for the breast cancer model if developed using particular sample sizes and based on particular assumed core models and available data

**Data used to inform sample size calculations,** **assumed core model**	**Sample size for new model development**	**95% uncertainty interval width:** *mean (min, med, max)*	**Mean absolute prediction error (MAPE):** *mean (min, med, max)*	**Probability of misclassification based on 20% threshold:** *mean (min, med, max)*
GBSG dataset of 220 patients, original core model (see the “Sample size calculation based on an existing dataset at hand” section)	355 (minimum no. recommended by *pmsampsize*)	0.23 (0.048, 0.22, 0.49)	0.046 (0.0066, 0.044, 0.11)	0.057 (0, < 0.0001, 0.48)
	920 (no. to target 95% uncertainty interval widths ≤ 0.2 in those with true risks ≤ 0.3)	0.14 (0.019, 0.13, 0.32)	0.029 (0.0032, 0.027, 0.066)	0.037 (0, 0, 0.47)
Synthetic dataset of 10,000 patients provided with predictor values & survival times, original core model (see the "Sample size calculation based on a synthetic dataset with follow-up information" section)	355	0.22 (0, 0.21, 0.56)	0.046 (0, 0.043, 0.12)	0.054 (0, < 0.0001, 0.50)
	920	0.14 (0, 0.13, 0.37)	0.029 (0, 0.028, 0.084)	0.037 (0, 0, 0.50)
Synthetic dataset of 10,000 patients provided with predictor values but without survival times, original core model (seethe "Sample size calculation based on a synthetic dataset without follow-up information" section)	355	0.22 (0, 0.21, 0.58)	0.046 (0, 0.044, 0.12)	0.055 (0, < 0.0001, 0.50)
	920	0.14 (0, 0.13, 0.39)	0.030 (0, 0.028, 0.081)	0.037 (0, 0, 0.50)

The anticipated 95% uncertainty intervals around individual risk are quite narrow, with a mean width of 0.23 and a mean MAPE of 0.046. The probability of misclassification (proportion of the uncertainty distribution below/above the threshold value of 20% when ‘true’ risk is actually above/below) is generally very low, with a mean probability of 0.057. Those with very high ‘true’ risks have a MAPE of about zero. However, MAPE is higher for individuals with ‘true’ risks ≤ 0.3, and some have misclassification probabilities over 0.1, even toward 0.5, with a mean of 0.18. Therefore, a sample size of 355 participants might be considered too low, if greater precision of risk estimates is needed for those with ‘true’ risks ≤ 0.3, especially as (e.g. chemotherapy) decisions for this group are very sensitive to understanding their actual risk.

This issue could be discussed via patient and public involvement and engagement (PPIE) groups and with clinical stakeholders, to identify target precision required, which may differ across the spectrum of ‘true’ risks from 0 to 1. Then, we can apply Eq. (11) to identify the sample size required to target the precision required. For example, consider that stakeholders recommend targeting 95% uncertainty interval widths < 0.20 for all individuals with a ‘true’ risk of ≤ 0.3 (Fig. 1b). Applying Eq. (11) identifies a total sample size of about 920 participants is needed to achieve this; this sample size would also reduce this subgroup’s anticipated mean misclassification probability down from 0.18 to 0.12. Note that the more stringent the precision and misclassification criteria required, the larger the sample size needed.

### Sample size calculation based on a synthetic dataset including follow-up information

Now let us consider that the GBSG dataset is not available, for example due to it being held externally and unavailable for transfer (e.g. due to confidentiality constraints or access restrictions). In that situation, researchers could ask the data holders to generate a large synthetic dataset to mirror the real dataset, in terms of the joint distribution of predictors, and censoring and recurrence times. This synthetic dataset could then be used in the sample size calculation. To illustrate this, we used the *synthpop* library in R [22], to produce a synthetic dataset of 10,000 participants with randomly generated values of the six core predictors alongside follow-up time and whether they were censored or had a recurrence at that time. Example code is provided in supplementary material S2. Briefly, this approach utilises the joint distribution of variables as carefully specified in terms of a series of conditional distributions, similar to how multiple imputation is undertaken using chained equations. Simulating a large dataset of 10,000 participants ensures (if the conditional distributions are carefully chosen) that the observed (marginal and conditional) distributions of variables in the original GBSG dataset will be closely reflected by the synthetic data (e.g. in terms of means and SDs of continuous variables; category proportions of categorical variables). This can be checked by the original data holders, before finalising their synthetic data to be passed over.

Once the synthetic data are obtained, the researchers can use this to apply the sample size calculation (and *pmstabilityss*) following the same steps as described earlier. Note that the large size of the synthetic data (10,000 participants) is simply to help ensure the distributions of predictors, and other variables closely reflect those in the (unseen) GBSG dataset; it is *not* the sample size required for model development. The synthetic dataset is used to generate the unit information matrix in Step (5), and then Step (6) examines the actual sample size required for model development based on this unit information matrix.

**Fig. 2:**
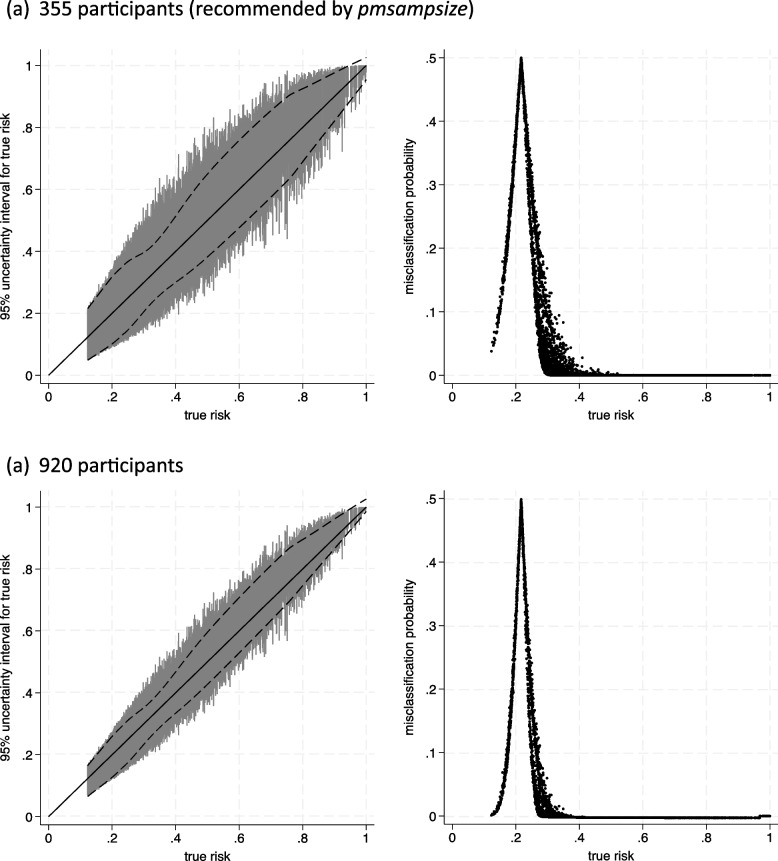
Prediction and classification instability plots for the breast cancer recurrence model assuming a particular sample size, using a synthetic dataset for informing the joint distribution of predictors and censoring and recurrence times** a** Three-hundred fifty-five participants (recommended by pmsampsize).** b** Nine-hundred twenty participants

The results based on the synthetic data are summarised in Table 1 and Fig. 2; they are very similar to when the GBSG dataset was available directly, with similar summary statistics about uncertainty interval widths and misclassification probabilities. For example, with 355 participants, the mean MAPE is 0.046 when using either dataset, and the mean uncertainty interval width is now 0.22 compared to 0.23 before. Hence, similar conclusions about the sample size required would be achieved in general, as is expected when the synthetic dataset is generated to closely match the characteristics of the original dataset.

Slight differences do arise in the lower range of predictions, potentially due to the larger size of the synthetic data providing more individuals with lower risks than before. In particular, the mean uncertainty interval width in those with ‘true’ risks ≤ 0.3 is slightly wider when using the synthetic data than when using the GBSG data directly, leading to a higher mean probability of misclassification (0.22 compared to 0.18 with 355 participants, 0.16 compared to 0.12 with 920 participants). Subsequently, larger sample sizes would be recommended based on the synthetic data if targeting uncertainty interval widths ≤ 0.2 in the subgroup with ‘true’ risks ≤ 0.3.

### Sample size calculation based on a synthetic dataset without follow-up information

Sometimes a (synthetic) dataset may be available for informing the joint distribution of predictors but without information about individual-level follow-up times and event status (e.g. due to confidentiality concerns). In this situation, implementing the sample size calculation requires the researcher to generate survival times under some sensible assumptions, including the anticipated maximum follow-up length, the mean follow-up time, and the censoring distribution.

To illustrate this, we revisit the sample size calculation using the synthetic dataset from the previous section but exclude any follow-up information. That is, the synthetic dataset now contains the values of the core predictors for the 10,000 participants so that we have the joint predictor distribution needed for Step (2). However, we do not have the value of ${w}_{i}=\text{exp}\left({y}_{i}+{\widehat{\mu }}_{i}\right)$ for each individual needed from Step (4). To address this, each individual’s ${\widehat{\mu }}_{i}$ is set to the ${\mu }_{i}$ from the ‘core model’ from Step (3), and we need to generate ${y}_{i}$, which is the minimum of the log event time and log censoring times for each patient. Using *survsim *[24], we generate the log event time for each patient conditional on their ${\mu }_{i}$ defined by the exponential regression of the ‘core model’; given the large dataset, this still ensures that the overall risk at 5 years was 0.39, akin to that for the GBSG dataset (example code in supplementary material S2). Then, we assume no censoring occurs in the first 2 years (based on the premise that drop out before 2 years is very unlikely), and between 2 and 7.28 years, there is uniform censoring, with all remaining individuals censored at 7.28 years (to match the maximum follow-up known for the GBSG). This led to the synthetic dataset having a mean follow-up time of 3.55 years across all participants (recall it was 3.57 years in the GBSG dataset).

**Fig. 3:**
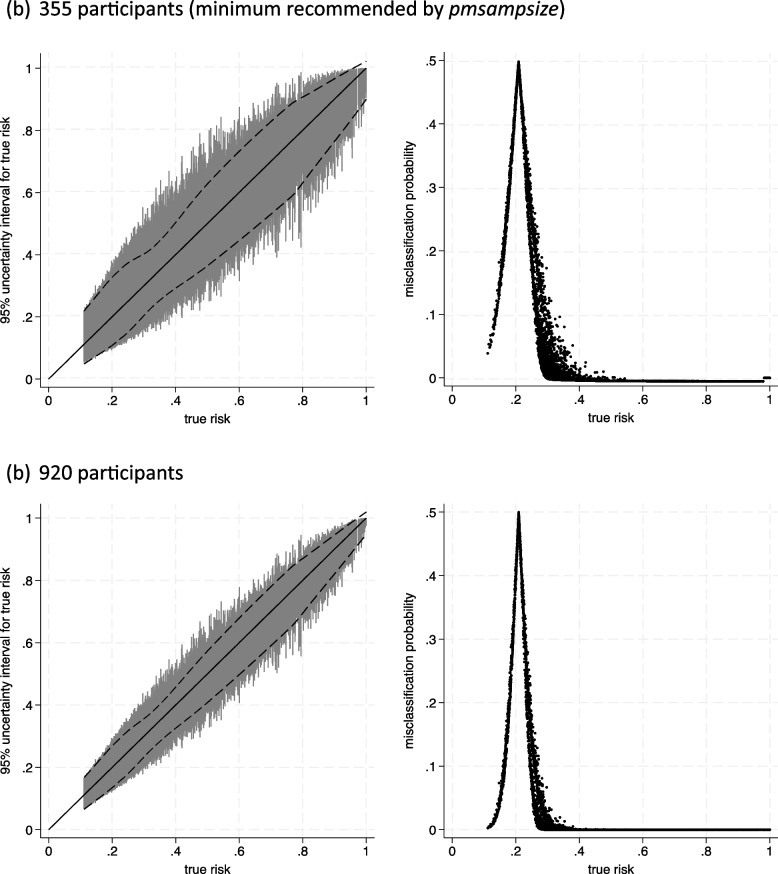
Prediction and classification instability plots for the breast cancer recurrence model assuming a particular sample size, using a synthetic dataset providing the joint distribution of predictors and then separately generating censoring and recurrence times **a** Three-hundred fifty-five participants (minimum recommended by pmsampsize).** b** Nine-hundred twenty participants

The results are summarised in Table 1 and Fig. 3 and are very similar to previous examples where follow-up times were directly available either within the GBSG dataset directly or the supplied synthetic dataset. For example, with 920 participants, the mean misclassification probability is 0.037, which is the same as previously. When considering just those with true risks ≤ 0.3, the mean misclassification probability is 0.15 compared to 0.12 when the GBSG dataset was available directly and 0.16 when the provided synthetic data included follow-up times.

## Results II: Examination of stability in subgroups to inform model fairness checks

Robustness of model predictions in subgroups may form part of fairness checks for using a model in practice and is a recommendation within the TRIPOD + AI reporting guideline [35]. Thus, as part of the sample size calculations, it may be important to examine anticipated uncertainty intervals and classification instability in subgroups defined by relevant patient characteristics. For this reason, in Step (1), we mentioned that the core predictors may include variables that represent protected characteristics. The model might include such predictors in the ‘core model’ or leave them out; regardless, precision and classification instability can be checked as long as the relevant variables are available after Step (1).

**Fig. 4:**
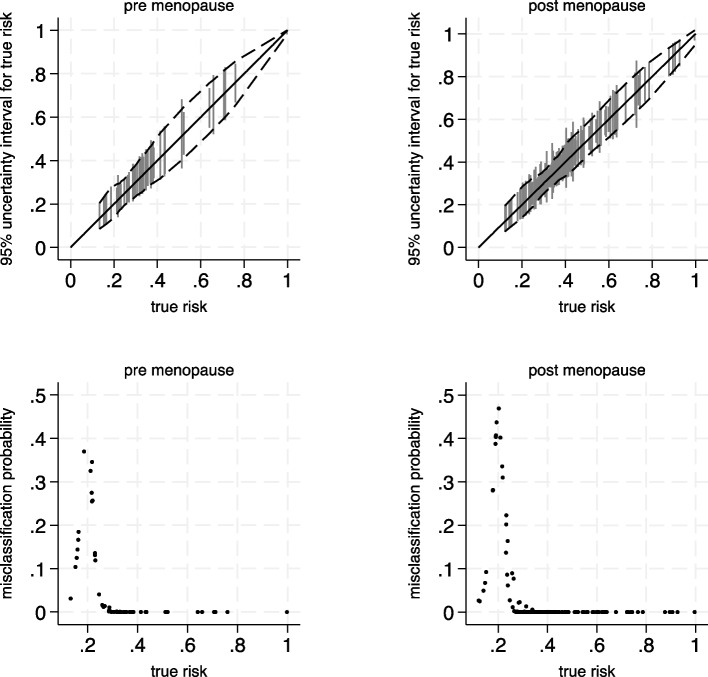
Prediction and classification instability plots for pre- and post-menopausal women when developing the breast cancer recurrence model assuming a total sample size of 920 participants

To illustrate this, we return to the breast cancer example and examine how a sample size of 920 participants is anticipated to impact subgroups defined by menopause status, as information from prediction models should be reliable for women both pre- and post-menopause. The results (based on having the GBSG dataset available) are shown in Table 2 and Fig. 4 and suggest that risk estimates will be more uncertain for premenopausal women, for example with a mean MAPE of 0.032 compared to 0.027 for postmenopausal women. This is anticipated as the minority (26%) of participants in the GBSG dataset are pre-menopausal. They also have a higher mean misclassification probability (based on the 20% threshold) of 0.059 compared to 0.030. Nevertheless, the discrepancies between groups are small, and both groups are anticipated to have quite low misclassification probabilities. Thus, we conclude that a sample size of 920 participants is unlikely to provide important fairness concerns in terms of the model’s precision and classification ability for different menopausal groups.

**Table 2:** Summary statistics for the expected precision and probability of misclassification for the AKI model when developed using 920 participants

**Menopause status**	**95% uncertainty interval width** *Mean (min, med, max)*	**Mean absolute prediction error (MAPE)** *Mean (min, med, max)*	**Probability of misclassification** *Mean (min, med, max)*
Pre-menopause	0.14 (0.019, 0.13, 0.32)	0.032 (0.003, 0.030, 0.064)	0.059 (0, 0.00039, 0.37)
Post-menopause	0.13 (0.084, 0.12, 0.20)	0.027 (0.0050, 0.025, 0.065)	0.030 (0, 0, 0.47)

## Results III: Empirical comparison of exponential, Weibull and flexible parametric models

As mentioned previously, specifying an exponential regression as the ‘core model’ is a pragmatic decision to facilitate closed-form solutions for the sample size calculation. An important question, therefore, is whether these also serve as a reasonable approximation in situations where the baseline hazard is not constant.

**Fig. 5:**
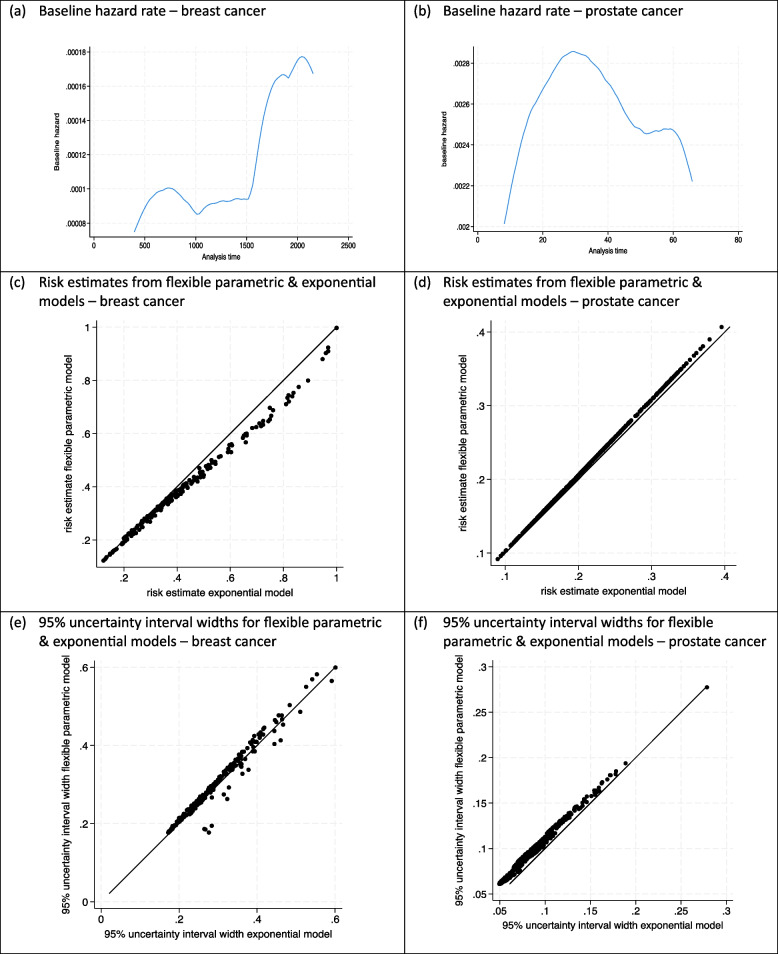
Baseline hazard rate for breast cancer and prostate cancer datasets (obtained by a weighted kernel smoother using ‘sts graph’ in Stata), with comparison of risk estimates and 95% uncertainty intervals when fitting an exponential or flexible parametric survival model

To examine this empirically, we fitted exponential, Weibull and flexible parametric regression models (with the same set of predictors as considered in the sample size calculation) to the existing breast cancer dataset of 220 participants, for which the baseline hazard appeared non-constant (Fig. 5a). The flexible parametric model used restricted cubic splines to model the baseline cumulative hazard [36, 37], with four internal knots. Post-estimation of each model, we derived 95% uncertainty intervals for the estimated risk of each of the 220 participants; these were calculated by using Eq. (7) to Eq. (10) for the exponential model (i.e. the same approach as in the sample size calculation) and by using the *predictnl* command in Stata for the Weibull and flexible parametric models (which numerically estimates standard errors and confidence intervals directly on the risk scale).

The estimated risks and uncertainty intervals are compared for the various models in Fig. 5 and supplementary material S4, which are generally quite similar for most participants. For example, the mean interval width is 0.291 for the Weibull model, slightly larger than the mean width of 0.286 for the exponential model. At the individual level, the largest disagreement in interval widths occurs in those with estimated risks close to 1, and in this situation, the *predictnl* standard errors and confidence intervals (on the risk scale) are themselves not robust for the Weibull and flexible parametric models, anyway.

We also compared the three approaches when applied to a prostate cancer dataset of 502 participants, to estimate 12-month mortality risk using four predictors. This example also showed a nonconstant baseline hazard (Fig. 5) but to a smaller degree than the breast cancer example. The risk estimates and uncertainty intervals for the three models were again very similar (Fig. 5, supplementary material S5), though the uncertainty intervals were slightly larger from the Weibull and flexible parametric models. We anticipate this will often be the case, due to the extra parameters being estimated.

In summary, these investigations give some reassurance that estimates of prediction uncertainty based on assuming an exponential core model is a good approximation for use in a sample size calculation.

## Discussion

In this article, we have considered the sample size required to develop a clinical prediction model using time-to-event (survival) data from a cohort study. We focused on targeting individual-level stability in risk estimates, building on previous work for binary outcomes [17]. Specifically, we proposed how to examine the (minimum) sample size required to produce individual risk estimates that are precise enough for the clinical context at hand. This can be done to inform the decision to collect new data (e.g. grant application for new model development or to set up a new cohort study or extend an existing one) or to help decide if an existing dataset is suitably large for model development. Our software *pmstabilityss* implements the approach and allows users to generate summary plots and statistics. Users might focus on one particular sample size (e.g. as defined by *pmsampsize* or an existing dataset being considered) or examine a range of sample sizes (e.g. for deciding the size of a new cohort study); alternatively, they could calculate the sample size required to achieve a chosen target mean uncertainty interval width, MAPE or misclassification probability.

When performing the calculations, the actual clinical setting of interest should be considered, with input from key stakeholders including patients and health professionals about acceptable levels of individual-level uncertainty and, if relevant, misclassification probabilities. Currently, such discussions between the model developers and stakeholders are either not done at all or tend to be conducted after model development. We hope our approach will motivate communication around this topic to begin at the onset of the development study, in the context of the clinical decision to be made and any relevant risk thresholds. Eliciting the key time-point(s) of interest for prediction is crucial, via discussion with patients, clinicians and other stakeholders [38]. In the breast cancer example, we focused on 5-year risk of recurrence, but sometimes multiple time-points may be of equal interest (e.g. 1 year, 5 year and 10 years), and the sample size required will change depending on the chosen time-point.

In our examples, we illustrated how to examine the anticipated precision of risks in key subgroups, to help inform fairness checks in the study design process. What constitutes an importance difference in precision of risks across subgroups is context specific. Clearly, differences in precision are expected across subgroups, and the uncertainty of minority groups will often be much larger than subgroups defined by more commonly observed characteristics. However, understanding this in advance may drive researchers to address this via a different sampling approach, such as purposefully sampling a larger proportion (than evident in the target population) of individuals from the minority groups, to help address concerns of imprecision or a lack of fairness. Such a sampling approach will distort the overall representativeness of the development dataset, however, and so it is important to then ensure that the developed model’s overall risk is recalibrated appropriately (e.g. by updating the intercept) for the target population of interest. Also, we recognise that precision of risk estimates across subgroups is just one aspect for informing fairness checks at the study design stage.

Our proposal utilises maximum likelihood estimation theory for unpenalised exponential regression models and uses Fisher’s information matrix to examine epistemic uncertainty (*reducible*model-based uncertainty) that arises from fitting an assumed ‘core model’ with a core set of predictors [39]. We do not consider aleatoric uncertainty (*irreducible* uncertainty) that refers to residual uncertainty that cannot be explained by the ‘core model’. The proposal also assumes parameter estimates will be unbiased (agree with the true ‘core model’ predictor effects on average). This is why we recommend starting at the minimum recommended by *pmsampsize*, which at least aims to minimise overfitting and aim for well-calibrated predictions at the population level. Also, unpenalised and penalised (shrinkage) approaches are likely to quite closely agree in this situation. Further research is needed to consider more complex settings, for example to deal with large numbers of candidate predictors (e.g. core predictors plus potential others, including noise variables), allowing variable selection, and penalised regression approaches like lasso. Other machine learning approaches, such as tree-based methods, may need substantially higher sample sizes to achieve the same level of stability compared to (penalised) regression approaches [40]. Again, further research is needed to examine this.

We assumed an exponential regression for simplicity, as it makes the ‘core model’ easier to prespecify with an assumed constant baseline hazard and facilitates closed-form solutions that are helpful for a sample size calculation. In two examples, the exponential model was a good approximation for quantifying uncertainty of risk estimates relative to either a Weibull model or flexible parametric model, even though the baseline hazard was not constant. Cox states that he generally prefers specifying survival models parametrically as ‘various people have shown that the answers are very insensitive to the parametric formulation of the underlying distribution’ [41].

The main obstacles are specifying the ‘core model’ and the joint predictor and censoring distributions. Choices may involve some subjectivity, and so sensitivity analyses may be warranted (as is often the case with any sample size calculation). We provided pragmatic suggestions to facilitate the process, for example by focusing on a small number of core predictors (and protected characteristics) of interest, by basing the ‘core model’ on previously published models or by assuming equal weighting of (standardised) predictors whilst adhering to a particular overall risk and C-index. Generally, our approach is more easily implemented when a synthetic or existing dataset is available (which is often the situation in practice), as then the joint distribution of core predictors can be observed directly, potentially alongside the censoring and event-time distribution.

Lastly, there is the potential for automating some or all of the sample size calculation steps by integrating them into existing software platforms that are designed to extract study datasets and process healthcare data, for instance the data extraction for epidemiological research (DExtER) tool [42]. Alternatively, they could be embedded alongside the production of synthetic datasets or at the point of obtaining feasibility information from large, anonymised longitudinal electronic healthcare record (EHR) databases (e.g. CPRD). This would facilitate and encourage appropriate sample size calculations by those using EHR data to develop prediction models.

In summary, we have proposed a new approach to inform the (minimum) sample size required for developing a clinical prediction model with a time-to-event outcome based on a ‘core model’ of established predictors. The approach enables researchers to examine how the sample size (for new data collection or an existing dataset) impacts individual-level uncertainty intervals and classification instability, to guide decisions on suitable datasets and sample size targets for model development.

## Supplementary Information

Supplementary File 1.

## Data Availability

A previous dataset is available of 686 patients diagnosed with node positive breast cancer from 1984 to 1989 and recruited to the German Breast Cancer Study Group (GBSG). We focused on the subset of 220 participants that were ER positive and received tamoxifen treatment. Data is available here https://hbiostat.org/data/
