# Supplementary material for: A decomposition of Fisher’s information to inform sample size for developing or updating fair and precise clinical prediction models — part 2: time-to-event outcomes
Source: Diagn Progn Res. 2025 Dec 16;9:33. doi: 10.1186/s41512-025-00204-9 (PMC12709744; doi:10.1186/s41512-025-00204-9)
Supplement: Supplementary file 1 [file 41512_2025_204_MOESM1_ESM.docx]

**SUPPLEMENTARY MATERIAL**

**S1: How to measure the impact of uncertainty on clinical utility**

Alongside the summary statistics and plots in the main paper, we can also measure the impact on clinical utility. If true risks (from the ‘core model’) ae known, then we have perfect information, and thus remove any prediction uncertainty and potential for misclassification, and maximise clinical utility from the core model. But with a particular sample size, the estimation error could reduce clinical utility, and we measure this as follow:

- *Expected loss in net benefit (plot and summary statistics).* Net benefit is a measure of clinical utility at a risk threshold ($z$) chosen for making decisions (e.g., initiating biopsy, starting treatment); for brevity we refer to explanations elsewhere.^31^ Each sampled value (e.g., 1000) of an individual’s estimated risk ($q_{i})$ from their prediction uncertainty distribution, leads to a sampled value of their loss (i.e. absolute difference) in net benefit ($d_{i}$) compared to knowing their true risk ($p_{i}$, from the core model) , where:

| $d_{i}=\left\{ \begin{matrix} 0 & if sign\left( q_{i}- z \right)=\mathrm{sign}\left( p_{i}- z \right) \\ \left\vert p_{i}-\left( \left( 1-p_{i} \right)\left( \frac{z}{1-z} \right) \right) \right\vert& \mathrm{otherwise} \end{matrix} \right.$ | Eq.(12) |
| --- | --- |

Essentially, $d_{i}$ is zero when the sampled estimated risk ($q_{i})$ and the true risk ($p_{i})$ are either both above or both below the threshold; otherwise $d_{i}$ is the absolute value of an individual’s contribution to the net benefit function. Finally, we can calculate,

| $Expected loss in net benefit for individual i= \Delta_{i}=E\left( d_{i} \right)$ | Eq.(13) |
| --- | --- |

which is estimated by their average sampled value of $d_{i}$, or equivalently

$$\Delta_{i}=E\left( d_{i} \right)={P(\mathrm{misclassification})}_{i}\times\left| p_{i}-\left( \left( 1-p_{i} \right)\left( \frac{z}{1-z} \right) \right) \right|$$

This can be plotted on a ‘Expected net benefit loss plot’, with $\Delta_{i}$ on the y-axis and $p_{i}$ on the x-axis. We can also sum $\Delta_{i}$ across all individuals, to estimate the expected total loss in net benefit in the population $(\Delta)$.

For the breast cancer example, the results obtained were as follows:

| **Data used to inform sample size calculations;**  **assumed core model** | **Sample size for new model development** | **Expected net benefit loss at 20% threshold**  *mean (min, med, max, sum)* |
| --- | --- | --- |
|  |  |  |
| GBSG dataset of 220 patients;  original core model  - see Section 4.1 | 355  (minimum no. recommended by *pmsampsize*) | 0.00003  (0, 0.00003, 0.015, 0.524) |
|  | 920  (no. to target 95% uncertainty interval widths ≤ 0.2 in those with true risks ≤ 0.3) | 0  (0, 0, 0.0093, 0.231) |

**S2: Example code for extended analyses**

1. **to generate a synthetic dataset from a real dataset in R**

# load the real dataset, load the synthpop module and choose a seed

library(synthpop)

myseed <- 66

# apply the synthpop module choosing the variables and their suitable assumed distributions

# here, the dataset only contains all the 7 variables of interest

# visit.sequence arranges the order of the variables

# first variable is always just a random sample of the original dataset so worth examining impact of this choice

# use minimumlevels to tell it which are factor variables

# k defines the size of the synthetic dataset

mysyn <- syn(myrealdata, minnumlevels = 3, method = c("norm", "lognorm", "polyreg", "lognorm", "lognorm", "logreg", "sample" ), k = 10000, visit.sequence = c(7, 1, 2, 3, 4, 5, 6), seed = myseed)

1. **using survsim in Stata to generate survival times**

*lambdas set to 0.0000975 based on trial and error to get S(5) to be about 0.39

set seed 560

survsim rectime , distribution(exponential) lambdas(0.0000925) covariates(LP_test 0.208)

gen censrec = 1

stset rectime, failure(censrec==1) scale(365.25)

sts graph

* with the original breast cancer data and swithing the event indicator to be 1 when they are censored

* we find that 10% censoring prob by 2 years, and then 70% by 5 years

* assume no censoring by two years but then uniform censoring afterwards with max follow-up of 7.2 years which is the assumed length of the new study like original GBSG daatset

gen censtime = (2*365.25) + runiform()*(5.28*365.25)

replace censrec = 0 if censtime < rectime

replace rectime = censtime if censtime < rectime

stset rectime, failure(censrec==1) scale(365.25)

* visualise the final KM curve

sts graph

* can check the censoring rate

*replace _d = (-1+_d)*-1

* sts graph

summ _t

streg age_sd size_sd nodes_sd g2 g3 meno , dist(exp) nohr

streg LP_test, dist(exp) nohr

**S3: Impact of changing risk threshold and predictor weights in the breast cancer example**

The required sample size depends heavily on the choice of risk threshold, which emphasises the need to establish relevant risk thresholds with stakeholders ultimately involved in the decision-making process. To illustrate this, consider that the designated risk threshold been much lower than 20%, say 5%; then the mean probability of misclassification is almost zero, even with a sample size of 355, as nearly all participants have ‘true’ risks much greater than 0.05. In that situation, larger sample sizes than suggested by *pmsampsize* may not be as necessary. Conversely, had the threshold been much higher, say 50%, then the mean probability of misclassification is 0.11 with 355 participants, about double that when using 20% threshold.

Let us now consider the impact of changing the ‘core model’ specification, for example to mimic a situation where less information is available about the relative predictor weights, and so pragmatic decisions are required. Recall in Section 3 we suggested, in the absence of more detailed information, to assume equal weights of *standardised* continuous predictors and set sensible weights for categorical variables. To examine this for the breast cancer model, we modified the ‘core model’ from Step (3) to be:

$$t_{i} \sim\mathrm{exponential}\left( \eta_{i} \right)$$

$${\ln\left( \eta_{i} \right)=\mu}_{i}$$

$$=\alpha+\delta\left( (-1 \times age) + (1 \times size) + (1 \times\#nodes) + (1 \times post\_menopause) + (1.5 \times grade2) + (3 \times grade3) \right)$$

This is labelled as the ‘alternative core model’. Standardised continuous predictors are each given the same weight, but the direction of effect is chosen to reflect previous knowledge that a younger age, a greater tumour size, and a higher number of nodes is associated with a higher recurrence risk. Furthermore, previous knowledge suggests post-menopausal women and a higher tumour grade are at increased risk, with post-menopause given the same weight as the continuous predictors, and tumour grades 2 and 3 assigned the highest relative weights (as grade is a well-known strong prognostic factor). We still assume prior information suggests an overall risk of 0.39 and C-index of 0.70 in the target population, and trial and error identifies that $\alpha$ and $\delta$ values of -2.911 and 0.230, respectively, ensure the ‘alternative core model’ achieve this in the GBSG dataset.

Applying our sample size approach, the prediction and classification instability plots are shown below. By changing the ‘core model’, the distribution of estimated risks changes, and there are fewer individuals with true risks below the 20% risk threshold. However, the magnitude of prediction and classification instability is quite similar to the original ‘core model’, as evident by very similar summary statistics for their uncertainty interval widths and MAPE values. For example, with a sample size of 355 participants, the mean uncertainty interval width is 0.23 for both core models, and MAPE is 0.046 and 0.047. As there are fewer individuals close to the 20% threshold, the mean misclassification probably is slightly lower than before, but otherwise changing the core model (though still retaining sensible weights) has not had a substantial impact. Clearly, the more discrepant the assumed core models, the more potential for differences; the same applies to assumptions about the joint predictor and censoring distributions.

1. 355 participants

1. 920 participants

**S4: Comparison of risk estimates and uncertainty interval widths from Weibull and exponential regression models, when applied to the breast cancer dataset of 220 participants**

**S5: Comparison of risk estimates and uncertainty interval widths for exponential and Weibull models applied to a prostate cancer dataset involving 502 participants and 4 predictors.**
